# Bacterial and parasitic contaminants of salad vegetables sold in markets in Fako Division, Cameroon and evaluation of hygiene and handling practices of vendors

**DOI:** 10.1186/s13104-018-3175-2

**Published:** 2018-02-06

**Authors:** Jane-Francis Tatah Kihla Akoachere, Bertrand Fossi Tatsinkou, Joseph Mbapngong Nkengfack

**Affiliations:** Department of Microbiology and Parasitology, Faculty of Science, University of Buea, PO Box 63, Buea, South West Region Cameroon

**Keywords:** Salad vegetables, Pathogenic bacteria, Antibiotic resistance, Intestinal parasites, Hygiene practices, Cameroon

## Abstract

**Objective:**

Increase in awareness of the health benefits of vegetables has resulted in an increase in consumption. Many vegetables are consumed raw to retain the natural taste and heat labile nutrients. The safety of raw vegetables is a great concern. We investigated the bacteriological and parasitological quality of salad vegetables sold in three major markets in Fako Division Cameroon, the hygiene and preservation practices of vendors and determined the antimicrobial sensitivity of bacterial isolates, to provide data that could be used to improve food safety and safeguard public health.

**Results:**

Bacterial contamination was high. Mean aerobic bacteria counts ranged from 2.5 × 10^6^ to 15 × 10^6^ cfu/g, total coliform counts from 4 to >  2400/g and fecal coliforms < 3 to 1100/g. Six bacterial species were isolated among which *Staphylococcus aureus* (35.4%) predominated while *Serratia marcescens* (8.5%) was the least. Bacteria showed high resistance to erythromycin (87.6%). Ten parasitic organisms were detected. *Balantidium coli* (25.6%) and *Entamoeba* spp. (21.7%) predominated. Contamination was highest in lettuce and lowest in green pepper. Hygiene and vegetable preservation practices of vendors were poor and could aggravate contamination. Contamination of fresh salad vegetables with pathogenic bacteria and parasites could be a food safety concern in study area.

**Electronic supplementary material:**

The online version of this article (10.1186/s13104-018-3175-2) contains supplementary material, which is available to authorized users.

## Introduction

Vegetables have health promoting characteristics [1, 2], being a source of vitamins and minerals, and phytochemicals some of which are antioxidant, phytoestrogens and anti-inflammatory agents. The recent increase in awareness of the health benefits of vegetables has resulted in increased consumption. Insufficient consumption of fruit and vegetable contributes to poor health and increases the risk of noncommunicable diseases [3]. Consumption of vegetables as part of a diet contributes to weight loss [4] reducing the risk of obesity, a risk factor for noncommunicable diseases. Because of their health benefits, WHO and FAO in 2003 launched a global initiative to promote the consumption of fruits and vegetables [5].

A lot of vegetables are consumed raw as salad to retain the natural taste and heat labile nutrients. The safety of vegetables eaten raw is a great concern as they have been shown to harbor pathogenic bacteria [6–10] and parasites [11–16]. Poor hygienic practices in the production and post-harvest system contribute to contamination [17, 18]. There are documented outbreaks of human infections associated with the consumption of raw vegetables [19]. Trade has contributed to geographic spread of these pathogens [20]. Recent years have witnessed an increase in the frequency of occurrence of these infections. Studies in developed and developing countries have demonstrated the potential of raw vegetables to transmit pathogens [8, 9, 21–23]. Data on microbial quality of vegetables in Cameroon is scarce despite the fact that it has witnessed an increase in vegetable consumption and cultivation, as cultivation in addition to ensuring food security, has become an income generating activity [17, 24]. Recent studies in Cameroon have reported the use of contaminated water for irrigation of vegetables [18, 25–28]. Thus, there is an urgent need to evaluate microbiological safety of salad vegetables sold in Cameroon. This study was aimed at investigating the microbiologic quality of salad vegetables from markets in Fako, Division Cameroon, to highlight their potential in disease transmission and provide data that could guide policy to improve food safety and safeguard public health. The hygiene and preservation practices of vendors were also investigated.

## Main text

### Methods

#### Study area and study design

The study was conducted in three towns in Fako, Cameroon: Buea, Tiko and Limbe. Buea, the capital of Southwest region is located on the eastern slopes of Mount Cameroon. According to data from the Cameroon Development Corporation (CDC) annual rainfall in Buea varies between 3000 and 3500 mm. Buea being the capital of former West Cameroon receives tourists who visit historic sites as well as Mount Cameroon.

Limbe is situated along the Atlantic coast of West Africa. It is bordered in the north by Buea, east by Tiko, west by Idenau and south by the Atlantic Ocean. Limbe and Buea have recently experienced an expansion in hotel and restaurant business as these towns have been hosting many national and international events.

Tiko is located 21 km from Limbe, and has a temperature range of 35–37 °C. Limbe and Tiko have warm equatorial climatic conditions. Humidity is as high as 80%.

Six vegetable types were purchased weekly from randomly selected vendors in three markets: Buea Central Market, Tiko Market and Limbe Market, for a period of 10 weeks from October to December 2015 and analyzed for bacteria and parasite contamination. The antimicrobial susceptibility of bacteria isolates and the hygiene and preservation practices of vendors were investigated.

#### Sample collection and processing

Thirty samples each of cucumber, carrot, lettuce, green pepper, green cabbage and red cabbage were purchased from randomly selected vendors. One sample of each vegetable was collected per week per site for 10 weeks (total of 180 samples). Each sample was placed in a sterile polythene bag and transported to the laboratory at temperature range 4–6 °C. Twenty-five grams of sample was immersed in 225 ml of sterile distilled water for 15 min, vigorously agitated and waste water used for analyses.

#### Enumeration of aerobic bacteria, total coliforms and fecal coliforms

Aerobic bacterial count was determined by the pour plate method using nutrient agar (Liofilchem^®^ s.r.l., Italy). A 10^−4^ dilution of waste water was prepared and 1 ml inoculated. Plates were incubated at 37 °C for 24 h.

Undiluted wash water was used for the enumeration of coliforms. Total and fecal coliforms were enumerated by the multiple fermentation test [29].

#### Isolation and identification of bacteria

The following media from Liofilchem^®^ s.r.l. (Italy) were used: nutrient agar, MacConkey agar, *Salmonella*-*Shigella* agar and mannitol salt agar. Agar plates were inoculated in duplicate with 100 µl of sample by the spread plate technique. Prior to inoculation of *Salmonella*–*Shigella* agar, samples were pre-enriched overnight in selenite F broth (Becton, Dickinson & Company) at 37 °C. Plates were incubated at 37 °C for 24 h. Pure cultures were characterized by Gram staining, motility, oxidase and catalase tests, and growth on triple sugar iron agar (Oxoid) [30]. Gram positive cocci were subjected to the coagulase test. The identity of gram negative rods was confirmed using the Analytical Profile Index 20E (Biomérieux SA, France) kit.

#### Antimicrobial susceptibility testing

The standard disc diffusion technique [31] was used. The following antibiotic discs from Oxoid (Basingstoke, England) were used: erythromycin (10 μg), gentamycin (30 μg), ampicillin (10 μg), ciprofloxacin (5 μg), chloramphenicol (30 μg) and streptomycin (10 μg). The diameters of inhibition zones were compared with those of the Clinical Laboratory Standards Institute (CLSI) [32].

#### Parasitological examination

Undiluted waste water was poured through sterile gauze and left for about 10 h to sediment. The supernatant was discarded and sediment centrifuged at 1207×*g* for 5 min. A drop of the sediment as well as iodine stained smears were examined microscopically. Identification was based on their morphology [33].

#### Evaluation of hygiene and preservation practices of vendors

A questionnaire was administered to 60 vendors (20 from each market), randomly selected from those samples were obtained. Data was also collected by visual inspection.

#### Data analysis

Data was entered in Epi Info version 3.5.3 and exported to SPSS version 20. The ANOVA test was used to compare differences in mean aerobic bacterial counts between various types of vegetables. The Bonferroni corrected post hoc *t* test (significance at P ≤ 0.0083) was used to compare the means of aerobic bacterial counts, total coliform counts and fecal coliform population of vegetables from various sites. The Chi square test was used to examine differences in the occurrence of bacterial isolates and parasites. Values were considered significant at P ≤ 0.05.

### Results

#### Aerobic bacterial, total coliform and fecal coliform load of samples

**Fig. 1:**
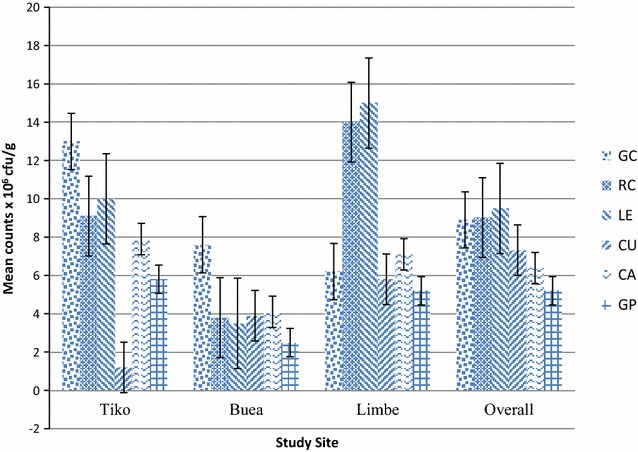
Mean aerobic bacterial counts (× 10^6^ CFU/g) of samples from study sites. Mean aerobic bacterial load of samples was generally high. Highest counts occurred in leafy vegetables green cabbage (GC), red cabbage (RC) and lettuce (LE) compared to non-leafy cucumber (CU), green pepper (GP) and carrots (CA). Number of each sample analyzed (n) = 30. ANOVA test gave a significant difference (P = 0.00) in counts between vegetables

Aerobic bacteria counts were high, ranging from 2.5 × 10^6^ to 15 × 10^6^ cfu/g. Lettuce had the highest mean count (9.5 × 10^6^ cfu/g) while green pepper had the lowest (5.2 × 10^6^ cfu/g) (Fig. 1). ANOVA test showed a significant difference in counts between vegetables (P = 0.00).

The overall mean total coliform count was highest in lettuce (1171.6 ± 117.16/g). In Tiko, mean counts were significantly higher in green cabbage (P < 0.0083) while in Limbe and Buea, counts were significantly higher in lettuce (Table 1).

**Table 1:** Mean (range) total coliform and fecal coliform load (MPN/g) of sample from various markets

Vegetable type	Tiko	Buea	Limbe	Mean counts
Total coliforms				
Green cabbage	1536 (240 to > 2400)	430.2 (93 to 1100)	1032.9 (93 to > 2400)	999.7 ± 1046.15
Red cabbage	618 (210 to > 2400)	199.5 (75 to 210)	1023.9 (63 to > 2400)	613.8 ± 914.84
Lettuce	618 (150 to > 2400)	1448.4 (21 to > 2400)	1448.4 (21 to > 2400)	1171.6 ± 117.16
Cucumber	154.2 (21 to 210)	152 (20 to 240)	790.0 (93 to > 2400)	365.7 ± 616.24
Carrot	1029.3 (21 to > 2400)	94.8 (21 to 210)	1041.9 (63 to > 2400)	722 ± 1081.44
Green pepper	102.9 (28 to 210)	103 (4 to 150)	306.3 (20 to 1100)	170.73 ± 264.34
Fecal coliforms				
Green cabbage	87.9 (4 to 210)	27.7 (20 to 39)	47.3 (7 to 93)	54.17 ± 66.55
Red cabbage	69.3 (9 to 210)	24.3 (9 to 39)	22.2 (11 to 28)	38.6 ± 58.86
Lettuce	115.5 (9 to 240)	291.1 (9 to 1100)	88.5 (21 to 240)	165.03 ± 270.95
Cucumber	59.7 (> 3 to 150)	67.3 (4 to 210)	273.5 (11 to 1100)	133.5 ± 272.79
Carrot	110.1 (9 to 210)	82.5 (15 to 240)	554.1 (4 to 1100)	248.9 ± 397.72
Green pepper	17.3 (9 to 28)	73.9 (11 to 210)	50.6 (4 to 39)	47.27 ± 62.86

*MPN* most probable number

Fecal coliforms were detected in all samples. Carrots and lettuce had highest count (248.9 ± 397.72 and 165.03 ± 270.95/g respectively). There were no significant differences in counts between vegetables (P ˃ 0.0083) (Table 1).

#### Bacteria isolated

**Fig. 2:**
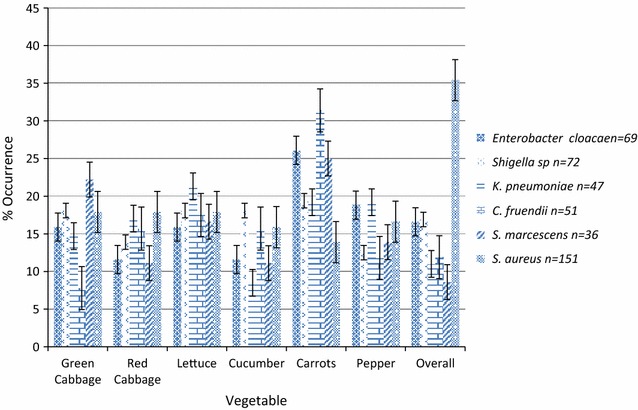
Distribution of bacteria isolates in samples. Six species of bacteria, mostly enteric organisms were detected in vegetables. *S. aureus*, the only non-enteric organism was the most frequently isolated. All six species were present in all types of vegetables. Only *C. freundii* (*X*^2^ = 16.118, P = 0.007) showed significant differences in the occurrence in various types of vegetables

Six bacteria species were isolated. *Staphylococcus aureus* (83.9%) was the predominant organism while *Serratia marcescens* (20%) was the least (Fig. 2). All six species were detected in all vegetable types. *S. aureus* was most frequently isolated from leafy vegetables while other bacteria were more frequently isolated from carrots.

#### Antimicrobial susceptibility of isolates

Ciprofloxacin (95.1%) was the most active drug. Resistance to erythromycin (75.6%) was highest. With the exception of 96 isolates of *Staphylococcus aureus* susceptible to erythromycin, all other bacteria were resistant (Additional file 1). One *Shigella* isolate was resistant to all antibiotics.

#### Contamination with parasites

Ten parasitic protozoans and helminths were detected*. Balantidium coli* (32.4%) was most frequently detected (Additional file 2). With the exception of *Trichuris trichiura*, all parasites were detected in Buea (Additional file 3). The highest level of contamination occurred in lettuce (25.4%) and the least in green pepper (8.5%) (Additional file 4). *Balantidium coli* and *Entamoeba* spp. were detected in all vegetable types.

#### Evaluation of hygiene and preservation practices of vendors

More females (76.5%) participated in the survey. The majority of participants (41.7%) had no formal education, and did not cultivate the crops (85%). Hand washing with soap was practiced by 23.3%. Only 16.7% had formal training on hygiene and food preservation (Additional file 5). Washing of vegetables was practiced by 35.0% amongst which 38.1% used stream water. None of the vendors transported vegetables to the market or sold them under controlled temperature conditions. Vegetables were placed on dirty bags during sale. Unsold vegetables were kept in the market (55.0%) or left in the backyard of their houses (45.0%).

### Discussion

Samples had high aerobic bacterial counts. Uzeh et al. [34] reported high levels of bacteria in raw vegetables. Bacterial counts were in the order: Lettuce > red cabbage > green cabbage > cucumber > carrot > green pepper. Foods are regarded as harmful when the bacterial load is high even if the bacteria are not known to be harmful [35]. Coliforms were detected in all samples. Mean counts showed a pattern similar to that observed for bacterial load. Lettuce leaves have a large surface area suitable for water, soil and air contact, and fecal droppings from birds making it more susceptible to contamination than the other vegetables. Green pepper is raised above the ground reducing contamination by soil bacteria. Its surface is smooth and surface area small limiting colonization. Highest fecal coliform counts were observed in carrots similar to the report of Weldezgina and Muleta [36]. Carrots being a root crop could have received contamination from the soil, irrigation water, animal wastes used as fertilizer, water used for washing and from handlers. Its pits and crevices retain dirt containing organisms which may not be easily removed by slight washing. Recent studies in Cameroon [25, 26, 28] report that these vegetables are irrigated with fecally polluted water. Six bacteria species were isolated with *Staphylococcus aureus* predominating. Apart from *Staphylococcus aureus,* all isolates are enteric organisms indicating fecal contamination. Similar bacteria have been isolated elsewhere [10, 37, 38]. Isolates were most sensitive to Ciprofloxacin and resistant to erythromycin. One isolate of *Shigella* was resistant to all six antibiotics tested. Antibiotic-resistant bacteria or resistance determinants are known to spread to humans via the food chain [39, 40]. Thus raw vegetables could be a source of multi-drug resistant pathogenic bacteria.

Ten species of intestinal parasites were detected. Surveys in other parts of the world have also shown that raw vegetables could be agents for transmission of these parasites [41]. *Balantidium coli* predominated similar to the report of Simon-Oke et al. [42]. Contrary to our findings, Alade et al. [43] reported a higher prevalence of *Ascaris lumbricoides*, while Olyaei and Hajivandi [44] reported *Toxocara leonine.* Similar to Olyaei and Hajivandi [44] we observed a low prevalence of *Fasciola* spp. and *Trichuris trichiura*. Leafy vegetables and carrots were more contaminated. Their uneven surfaces facilitate microbial attachment. Similar to Abougraina et al. [45] and Mohamed et al. [14], the highest level of parasite contamination was in lettuce and least in vegetables with smooth surfaces.

Our study showed a higher level of contamination (61.6%) with intestinal parasites than 13.5% in Khartoum [14], 36% in Ghana [46], and 58% in Tripoli [45]. However, a higher rate of contamination (75.9%) was reported in Kenya [47]. The detection of enteric bacteria and parasites in samples implies that they could be contaminated with enteric viruses. The majority of the participants do not practice good hygiene thus increasing the chances of contamination. Transportation to the market was under unhygienic and uncontrolled temperature conditions and this favour the growth of microorganisms [48]. Our study shows that there is an urgent need to sensitize vendors on good hygiene and preservation of vegetables, and the public on proper washing and sanitization of vegetables prior to consumption.

### Limitations

We did not investigate for the presence of other foodborne pathogens such as *Listeria monocytogenes, E. coli* O157:H7 as well as enteric viruses. We did not include a control strain in susceptibility testing. Also, the minimum inhibitory concentration (MIC) of potent antibiotics was not investigated.

## Additional files

**Additional file 1.** Antimicrobial susceptibility of bacteria isolates. Bacteria isolated were tested against six antibiotics of different classes. Ciprofloxacin was the most active drug while resistance to erythromycin was highest.

**Additional file 2.** Occurrence of Parasites in samples. Ten species of parasites at various developmental stages were detected. *Balantidium coli* was the most frequently detected while *Trichuris trichuria* was the least.

**Additional file 3.** Distribution of parasites in study sites. Not all ten species were detected in each study site. *Ascaris lumbricoides*, hookworm, *Strongyloides stercoralis, Balantidium coli* and *Entamoeba* species were detected in all study sites.

**Additional file 4.** Distribution of parasites in vegetables. All parasites were not detected in all types of vegetables. Only *B. coli* and *Entamoeba* species were found in all types of vegetables.

**Additional file 5.** Characteristics of vendors and hygiene and preservation practices. Hygiene and preservation practices of vendors was poor and this could aggravate contamination.

## Abbreviations

WHO: World Health Organisation
FAO: Food and Agricultural Organisation
CDC: Cameroon Development Cooperation
APHA: American Public Health Association
CFU: colony forming units
MPN: most probable number
