# Supplementary material for: Bacterial and parasitic contaminants of salad vegetables sold in markets in Fako Division, Cameroon and evaluation of hygiene and handling practices of vendors
Source: BMC Res Notes. 2018 Feb 6;11:100. doi: 10.1186/s13104-018-3175-2 (PMC5801804; doi:10.1186/s13104-018-3175-2)
Supplement: Supplementary file 5 — Additional file 5. Characteristics of vendors and hygiene and preservation practices. Hygiene and preservation practices of vendors was poor and this could aggravate contamination. [file 13104_2018_3175_MOESM5_ESM.docx]

**Additional File 5: Characteristics and hygiene practices of Vendors**

| Characteristics | Status | Number (%) |
| --- | --- | --- |
| Demography  Gender | Male | 14 (23.3) |
|  | Female | 46 (76.5) |
| What is your highest level of education | No formal education | 25 (41.7) |
|  | Primary | 12 (20.0) |
|  | Secondary | 23 (38.3) |
| Hygiene and handling practices of vendors | | |
| Do you washing your hands with soap? | Yes | 14 (23.3) |
|  | No | 46 (76.7) |
| Have you ever received any training on hygiene? | Yes | 10 (16.7) |
|  | No | 50 (83.3) |
| Where do you get your vegetables from? | Cultivated | 9 (15.0) |
|  | Purchase | 51 (85.0) |
| How do you package vegetables before transportation? | Unwashed bags | 20 (33.3) |
|  | Dirty baskets | 33 (55.0) |
|  | Washed bags | 1 (1.7) |
|  | Clean basket | 6 (10.0) |
| Do you transport these vegetables to the market under refrigeration conditions? | No | 60 (100) |
|  | Yes | 0 (00) |
| Do you wash vegetables before selling? | Yes | 21(35.0) |
|  | No | 39 (65.0) |
| If yes, where do you obtain after for washing vegetables? | Stream | 8 (38.1) |
|  | Pipe borne | 13 (61.9) |
| Do you sell all vegetables same day? | No | 38 (63.3) |
|  | Some times | 22 (36.7) |
| How do you preserve left overs? | In market | 33 (55.0) |
|  | At backyard | 27 (45.0) |
|  | In refrigerator | 0 (0.0) |
