# Supplementary material for: Bacterial and parasitic contaminants of salad vegetables sold in markets in Fako Division, Cameroon and evaluation of hygiene and handling practices of vendors
Source: BMC Res Notes. 2018 Feb 6;11:100. doi: 10.1186/s13104-018-3175-2 (PMC5801804; doi:10.1186/s13104-018-3175-2)
Supplement: Supplementary file 3 [file 13104_2018_3175_MOESM2_ESM.docx]

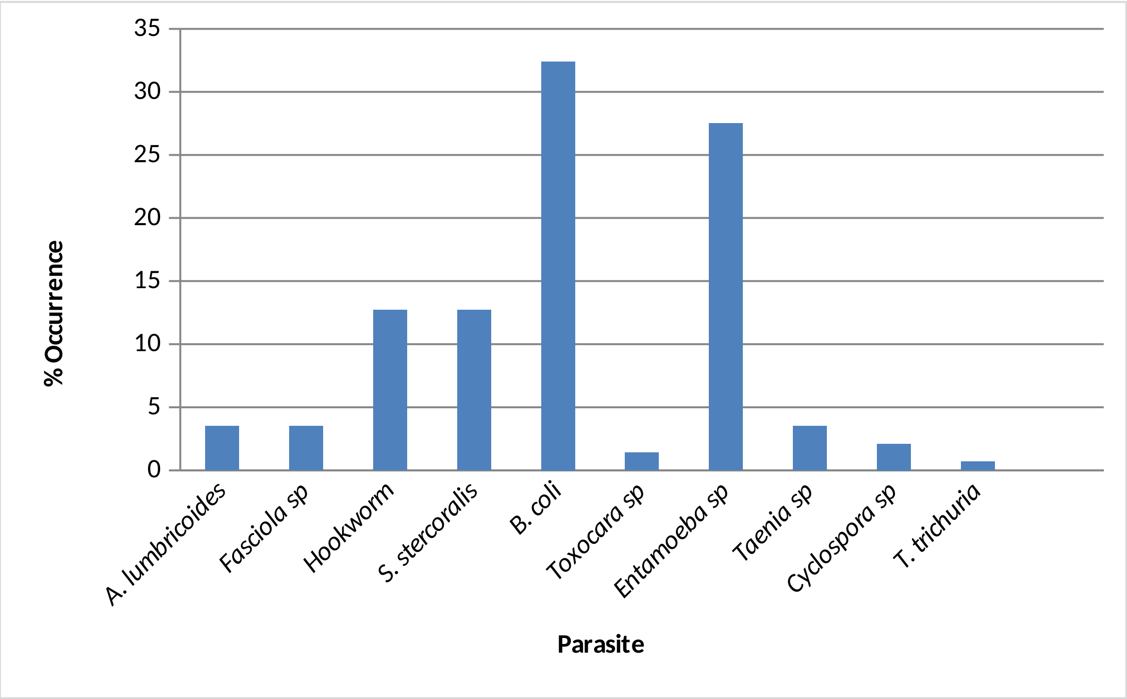
% Occurrence
35
30
25
20
15
10
5
0
A. lumbricoides
Fasciola sp
Hookworm
S. stercoralis
B. coli
Toxocara sp
Entamoeba sp
Taenia sp
Cyclospora sp
T. trichuria
Parasite
